# The *Gongora gibba* genome assembly provides new insights into the evolution of floral scent in male euglossine bee–pollinated orchids

**DOI:** 10.1093/g3journal/jkae211

**Published:** 2024-09-04

**Authors:** Maria Fernanda Guizar Amador, Kathy Darragh, Jasen W Liu, Cheryl Dean, Diego Bogarín, Oscar A Pérez-Escobar, Zuleika Serracín, Franco Pupulin, Santiago R Ramírez

**Affiliations:** Department of Evolution and Ecology, University of California, Davis, Davis, CA 95616, USA; Department of Evolution and Ecology, University of California, Davis, Davis, CA 95616, USA; Department of Evolution and Ecology, University of California, Davis, Davis, CA 95616, USA; Department of Evolution and Ecology, University of California, Davis, Davis, CA 95616, USA; Lankester Botanical Garden, University of Costa Rica, P.O. Box 302-7050, Cartago 30109, Costa Rica; Evolutionary Ecology Group, Naturalis Biodiversity Center, 2333 CR Leiden, The Netherlands; Lankester Botanical Garden, University of Costa Rica, P.O. Box 302-7050, Cartago 30109, Costa Rica; Royal Botanic Gardens, Kew, Richmond, Surrey TW9 3AE, UK; Herbario UCH, Universidad Autónoma de Chiriquí, P.O. Box 0427, David, Chiriquí 0427, Panamá; Lankester Botanical Garden, University of Costa Rica, P.O. Box 302-7050, Cartago 30109, Costa Rica; Department of Evolution and Ecology, University of California, Davis, Davis, CA 95616, USA; Lankester Botanical Garden, University of Costa Rica, P.O. Box 302-7050, Cartago 30109, Costa Rica

**Keywords:** genome assembly, *Gongora gibba*, chemotype A, orchid, floral scent, terpene synthesis

## Abstract

Orchidaceae is one of the most prominent flowering plant families, with many species exhibiting highly specialized reproductive and ecological adaptations. An estimated 10% of orchid species in the American tropics are pollinated by scent-collecting male euglossine bees; however, to date, there are no published genomes of species within this pollination syndrome. In this study, we present the first draft genome of an epiphytic orchid from the genus *Gongora*, a representative of the male euglossine bee–pollinated subtribe Stanhopeinae. The 1.83-Gb de novo genome with a scaffold N50 of 1.7 Mb was assembled using short- and long-read sequencing and chromosome capture (Hi-C) information. Over 17,000 genes were annotated, and 82.95% of the genome was identified as repetitive content. Furthermore, we identified and manually annotated 26 terpene synthase genes linked to floral scent biosynthesis and performed a phylogenetic analysis with other published orchid terpene synthase genes. The *Gongora gibba* genome assembly will serve as the foundation for future research to understand the genetic basis of floral scent biosynthesis and diversification in orchids.

## Introduction

Plant–pollinator interactions are thought to have played a key role in the diversification of flowering plants (Van der Niet *et al*. 2014). Orchidaceae is a particularly species-rich group, consisting of over 30,000 species and 880 genera (Dressler 2005; Chase *et al*. 2015; Christenhusz and Byng 2016; Govaerts *et al*. 2021; Pérez-Escobar *et al*. 2024). Orchids exhibit a wide range of highly specialized ecological and reproductive strategies, some of which are thought to contribute to their elevated diversification rates (Cozzolino and Widmer 2005; Xu *et al*. 2012). Multiple factors related to pollination, such as the evolution of deceptive pollination strategies and pollinator specialization, have been suggested to play a role in enhancing diversification rates (Givnish *et al*. 2015; Ackerman *et al*. 2023).

A striking example of a reproductive strategy linked to higher diversification rates is pollination by euglossine bees (or orchid bees; Apidae: Euglossini; Dressler 1968; Gerlach and Schill 1991; Ramírez *et al*. 2011; Givnish *et al*. 2015). Fragrance is thought to be the second most common reward among orchids, predominantly associated with euglossine bee pollination (Ackerman *et al*. 2023). In this pollination system, male bees pollinate plants while visiting inflorescences to collect chemical compounds, which they store in hind-leg pockets for later use acting as a pheromone analog during courtship display (Allen 1954; Eltz *et al*. 2005; Henske *et al*. 2023). The production of floral scent not only attracts pollinators but is itself the reward. Therefore, all *Gongora* species lack additional floral rewards, such as nectar.

*Gongora*, one of at least 22 genera that exhibit euglossine bee pollination, contains 60–70 recognized species. However, the taxonomy and the identification of species in the genus *Gongora* are notoriously difficult because multiple cryptic species, with little morphological variation, can coexist, and are only discernible by their floral scent (Dressler 1966; Whitten 1985; Jenny 1993; Hetherington-Rauth and Ramírez 2016; Guizar Amador 2022). *Gongora* emit species-specific floral scents, typically consisting of a few main compounds alongside minor compounds. Differences in the floral scent between chemotypes, or distinct chemical groups, lead to the attraction of different pollinators, which is thought to maintain reproductive isolation barriers (Guizar Amador 2022). Pollinator-driven diversification is hypothesized to have played a major role in the evolutionary history of *Gongora* (Dressler 1968; Williams and Dodson 1972; Ramírez *et al*. 2011); however, the molecular and genetic mechanisms underlying the origin and maintenance of reproductive barriers remain largely unexplored.

Generating genomic resources for *Gongora* is needed to elucidate the genetic basis of floral scent production and reveals how divergent floral scent phenotypes evolve and lead to reproductive isolation. To date, no reference genomes are available for any euglossine bee–pollinated orchids, a major obstacle toward studying the diversification of this group. In this study, we report the genome of *Gongora gibba*, a member of the *Gongora* subgenus previously described as chemotype A (Supplementary Fig. 1;Hetherington-Rauth and Ramírez 2016; Guizar Amador 2022). We report the assembly size, the repeat sequences detected, as well as an annotation. We confirm the identity of *G. gibba* by placing this species in a plastid phylogeny. We also conducted a high-quality annotation of terpene synthase (TPS) genes, laying the foundation for further research on floral scent biosynthesis.

## Materials and methods

### Sample preparation and sequencing

All plant materials used for the genome assembly were obtained from a mature *G. gibba* plant collected from the surroundings of the La Gamba Tropenstation in the province of Puntarenas, located in southwestern Costa Rica (BioSample no. SAMN37328957, BioProject no. PRJNA1014482). The sample was imported to the United States under the CITES Certificate of Scientific Exchange permit no. 14US51372B/9 and is currently located in the Botanical Conservatory at the University of California, Davis (ID: G-10, Supplementary Fig. 1b). For genome sequencing, we collected fresh leaves and the leaves were flash-frozen in liquid nitrogen. Two tissue samples were shipped to Dovetail Genomics (Santa Cruz, CA, USA) for the construction and sequencing of 2 Illumina libraries (Illumina HiSeq 2500 and NextSeq 2000, insert sizes 402 and 523 bp, respectively), 1 PacBio Sequel library (5 SMRT cells, 7,876 bp average read length), 1 Hi-C library (Illumina HiSeq X), and 1 Chicago library (Illumina HiSeq X). An additional sample was used for DNA extraction with a DNEasy Plant Mini Kit from Qiagen, followed by library construction and sequencing using an Illumina HiSeq 4000 platform. In total, we generated 412 Gb of raw reads that were then filtered based on sequencing quality and adapter contamination.

### Genome size estimation

To estimate a genome size and a heterozygosity of *G. gibba* plant, we analyzed the *k*-mer frequency distribution from the 402-bp insert size Illumina library with Jellyfish (Marçais and Kingsford 2011). We also estimated the genome size of the plant with flow cytometry. Briefly, a 1.5-cm^2^ fresh orchid leaf was chopped with a fresh single-edge razor blade in a cold Galbraith buffer along with a similar-sized fresh leaf from either tomato (*Solanum lycopersicum*, 1C = 1,320.3) or pea (*Pisum sativum*, 1C = 4,591.71). The released nuclei were then filtered and stained with a cold solution of 25 mg/mL propidium iodide for 30 min in the dark. We quantified the relative fluorescence of 2C orchid and 2C standard nuclei using a Beckman Coulter CytoFLEX flow cytometer. A ploidy level was determined by the relative position of the 2C orchid and 2C standard peaks and by the estimated genome size based on the ratio of the 2C peak positions of the sample and standard times the amount of DNA in the standard.

### Genome assembly

Given the high levels of heterozygosity and repetitive content in the *G. gibba* genome, we decided to use a hybrid strategy for the assembly. Long reads can improve the contiguity of an assembly; however, this technology is associated with high error rates (Zhang *et al*. 2020). We used FMLRC (Wang *et al*. 2018) with one of our Illumina libraries (SRCD2 S1 L001) to leverage the higher accuracy of the short reads to perform long-read error correction on the PacBio library. Before proceeding with the assembly, we used BLAST (Altschul *et al*. 1990) to identify long reads not belonging to the nuclear genome by comparing them against the *Oncidium* plastid (GQ324949.1) and mitochondrial (KJ501920.1) sequences downloaded from the NCBI (Wheeler *et al*. 2007). The resulting 85,553 reads were removed from the nuclear genome assembly and used separately to assemble the organelle genomes.

With the corrected and filtered PacBio reads as input, we used WTDBG2 (Ruan and Li 2020) for the de novo assembly. One of the short-read libraries (SRCD2 S1 L00) was then mapped to the contigs using BWA (Li 2013; Supplementary Table 1). Contigs with different levels of coverage were searched using the BLAST against the NCBI's nucleotide library to determine whether they belong to exogenous DNA. Contigs with >70% of their length are not covered by any Illumina reads that matched bacterial DNA, so 70% was established as a cutoff point to remove exogenous contigs from the assembly.

SSPACE v3.0 (Boetzer *et al*. 2011) and the 523-bp insert size Illumina library were used for a preliminary scaffolding step. To improve the accuracy of the assembly, pilon (Walker *et al*. 2014) and the 402-bp insert size library were then used to polish the assembly. This preliminary assembly was sent to Dovetail Genomics for further scaffolding with the Chicago and Hi-C libraries, which were used as input data for HiRise, a software pipeline designed for utilizing proximity ligation data to scaffold genome assemblies (Putnam *et al*. 2016). Completeness of the genome assembly was assessed using Benchmarking Universal Single-Copy Orthologs (BUSCO; Simão *et al*. 2015) with default parameters and the embryophyta dataset.

All raw sequence data and the final genome assembly were deposited under the BioProject accession no. PRJNA1014482. All details of parameters used in the genome assembly are given in the supplementary file Genome_Assembly_Report.ipynb available at OSF (https://osf.io/hqav3/?view_only=5d65957cc6474488b53847219a7b6ac4).

### Plastid sequencing, assembly, and annotation of other *Gongor*a species

Using the BLAST, we identified *G. gibba* scaffolds that matched the *Oncidium* organelle genomes. These scaffolds, and the previously identified chloroplast and mitochondrial PacBio reads, were used as an input for Canu (Koren *et al*. 2017) to perform de novo assemblies. SSPACE v3.0 (Boetzer *et al*. 2011), pilon (Walker *et al*. 2014), and GetOrganelle (https://github.com/Kinggerm/GetOrganelle) were used to improve the contiguity and accuracy of the assemblies.

The plastid genomes of 10 additional *Gongora* samples from Costa Rica and Panama were assembled to determine the phylogenetic position of the sequenced *G. gibba* (A-chemotype). Total DNA was extracted from the fresh leaves dried in silica gel using the CTAB method (Doyle and Doyle 1987). Library preparation and sequencing were performed at Genewiz GmbH (Leipzig, Germany) using the NovaSeq 6000 sequencing system. Paired-end reads of 150 bp were obtained for fragments with an insert size of 300–600 bp. Raw sequences were quality-filtered using Trim Galore v.0.6.5 (https://www.bioinformatics.babraham.ac.uk/projects/trim_galore/) with a Phred quality threshold of 30 (-q 30 –paired) and a minimum read length of 20 (−length). De novo assembly of the plastid genomes was performed for each sample using GetOrganelle (https://github.com/Kinggerm/GetOrganelle; Jin *et al*. 2020).

The assembled plastid genomes were annotated with the GeSeq application (Tillich *et al*. 2017) in MPI-MP CHLOROBOX (https://chlorobox.mpimp-golm.mpg.de/index.html). Multigene alignments of all plastid genomes were constructed using the HomBlocks pipeline (Bi *et al*. 2018). Phylogenetic trees were reconstructed using maximum likelihood with RAxML-8.2.4 (Stamatakis 2014). Bootstrap percentages were calculated using 1,000 replicates to assess node support. The *Erycina pusilla* (L.) N.H. Williams and M.W. Chase (JF746994) plastid genome was selected as the outgroup, retrieved from the NCBI GenBank (https://www.ncbi.nlm.nih.gov/genbank/). The resulting trees were visualized in FIGTREE v 1.4.4 software. The plastid raw reads were deposited under BioProject no. PRJNA1146482.

### RNA-seq for gene annotation

To generate RNA-seq data for *G*. *gibba*, we collected 1 root tip, 1 young pseudobulb, and 1 young inflorescence in the day (between 8 and 9 Am), in addition to 8 floral samples, consisting of 3 hypochiles (a basal portion of labellum) and 3 epichiles (an apical portion of labellum) sampled during the day and 2 hypochiles sampled at night (9 Pm). The tissue samples were placed in 2-mL centrifuge tubes filled with two 3-mm glass beads and immediately flash-frozen in liquid nitrogen. The tissue was disrupted using a Mixer Mill MM400 (Retsch), using 3 rounds of 30-s homogenization at 30 Hz, with cooling in liquid nitrogen between runs. RNA was then extracted using a standard RNeasy Mini protocol with QIAshredder following manufacturer's instructions, using a final elution with 30 μL of water. RNA was freeze-dried in GenTegra tubes and rehydrated just before sending for sequencing, following the manufacturer's instructions. The samples were sent to Novogene for quality control, library preparation, and sequencing. The samples were sequenced using 150-bp paired-end reads on a NovaSeq 6000. This generated ∼47 million reads per library (mean = 47.17 million, SD = 4.29 million, *n* = 11). The raw sequence data were deposited in the SRA under the BioProject accession no. PRJNA1027883. We trimmed the reads using Trim Galore! (Martin 2011). We then mapped the reads to the *G. gibba* genome assembly using 2-pass mapping in STAR (Dobin *et al*. 2013). We concatenated the BAM file to use in the annotation as described below.

### Repeat annotation

Tandem repeats and transposable elements were identified and annotated using the Extensive de novo TE Annotator (EDTA) v1.9.8 pipeline (Ou *et al*. 2019), RepeatModeler v.2.0.1 (Flynn *et al*. 2020), and RepeatMasker v.4.1.2 (Tarailo-Graovac and Chen 2009). Briefly, the EDTA pipeline and RepeatModeler were used for both ab initio and homology-based identification of TEs and tandem repeats, producing *G. gibba* repeat libraries. These libraries were combined using USEARCH to cluster sequences with >80% identity and remove all but 1 sequence from each cluster (Edgar 2010). We then used RepeatMasker with the custom library (Gongorav1_lib1.fa) to generate a masked genome (Gongorav1.fa.masked). Custom library and masked genome files can be found at the OSF (https://osf.io/hqav3/?view_only=5d65957cc6474488b53847219a7b6ac4).

### Gene prediction

We annotated the *G. gibba* genome using BRAKER3 (braker.pl v3.0.2) which combines RNA-seq and protein data in an automated pipeline (Lomsadze *et al*. 2005, 2014; Stanke *et al*. 2006, 2008; Gotoh 2008; Iwata and Gotoh 2012; Buchfink *et al*. 2015; Hoff *et al*. 2016, 2019; Kovaka *et al*. 2019; Brůna *et al*. 2020, 2021; Pertea and Pertea 2020; Gabriel *et al*. 2023). Along with the masked genome, we provided BRAKER with the RNA-seq data generated as described above and plant proteins downloaded from OrthoDB (odb10_plants; Zdobnov *et al*. 2021). In this pipeline, GeneMark-ETP was trained using the RNA-seq and protein hints, and then AUGUSTUS (Stanke *et al*. 2006, 2008) was run on the GeneMark-ETP prediction and also predicts a set of genes. TSEBRA (Gabriel *et al*. 2021) was then used to combine the predictions of AUGUSTUS and GeneMark-ETP to preserve only the genes with the highest evidence. The BUSCO (Simão *et al*. 2015) and OMArk (Nevers *et al*. 2022) were used to evaluate the completeness and consistency of the final set of gene models. We transferred the annotation to the final genome version after contaminant removal by the NCBI using Liftoff (Shumate and Salzberg 2021).

### Annotation of TPS genes

To improve the annotation of TPS genes, we used 2 different pipelines. First, we used bitacora (Vizueta *et al*. 2020), a pipeline that curates an existing annotation and finds additional gene family members. To do this, BLASTP and hmmer were used to search the existing annotation for gene family members of interest (Altschul *et al*. 1990; Eddy 2011). Then, additional regions of the genome were searched using TBLASTN, annotated using GeMoMa (Keilwagen *et al*. 2019), and validated with hmmer to create a new set of genes to add to the originally annotated set. As input, we used 2 HMM profiles: Terpene_syth_C (PF03936) and TPS N-terminal domain (PF01397) downloaded from Pfam. In addition, we provided previously annotated genes from *Arabidopsis thaliana* and *Oryza sativa* (Chen *et al*. 2011; Yu *et al*. 2020; Jia *et al*. 2022). In addition, we searched the *G. gibba* genome with the same set of protein sequences using TBLASTN (Altschul *et al*. 1990) and then annotated proteins on these scaffolds using exonerate (Slater and Birney 2005). Gene models from both bitacora and exonerate, and genome-wide annotations from BRAKER, were compared and TPS genes were manually curated using IGV (Robinson *et al*. 2011). We also created an annotation based on the concatenated RNA-seq bam file using StringTie (Pertea *et al*. 2016) to compare with our other annotations in IGV (Robinson *et al*. 2011). To check the final set of TPS genes for complete protein domains, we used the NCBI conserved domain search (Marchler-Bauer *et al*. 2015). All TPS gene annotations were then merged with the genome-wide annotation file (Gongora_annotation.gff3). Sequences for all TPS genes are also available (TPS_dna.fa, TPS_aa.fa). Those sequences considered pseudogenes were not included in the following phylogenetic analyses (TPS_psuedo.fa). All files are available at the OSF (https://osf.io/hqav3/?view_only=5d65957cc6474488b53847219a7b6ac4).

The predicted *G. gibba* TPS protein sequences, along with those from *A. thaliana* and *O. sativa*, were combined with TPS sequences from other orchids (*Apostasia shenzhenica*, *Dendrobium catenatum*, *Phalaenopsis aphrodite*, *Phalaenopsis equestris*, and *Vanilla planifolia*; Yu *et al*. 2020; Huang, Huang, *et al*. 2021) and aligned with MAFFT v7.508 (-maxiterate 1000, using L-INS-I algorithm; Katoh and Standley 2013). We then trimmed the alignment using trimAl (-gt0.6, sites only included when present in 60% of sequences; Capella-Gutierrez *et al*. 2009). Based on this alignment, we reconstructed a gene tree using 198 sequences in IQ-TREE with the ModelFinder function to determine the best-fit model (1,000 bootstraps, model JTT + F + G4; Nguyen *et al*. 2015; Kalyaanamoorthy *et al*. 2017; Hoang *et al*. 2018). We rooted the tree using the midpoint.root function in the phytools package in R (Revell 2012; R Core Team 2023). This midrooted version was then plotted using the following packages in R: ape (Paradis and Schliep 2018), evobiR (Blackmon and Adams 2015), ggtree (Yu *et al*. 2017), and ggtreeExtra (Xu *et al*. 2021). All files for analysis are available at the OSF (https://osf.io/hqav3/?view_only=5d65957cc6474488b53847219a7b6ac4).

## Results

### Genome assembly

In this study, we reported the first draft of the *G. gibba* genome assembly (Supplementary Fig. 1). To overcome the high repeat content and heterozygosity, our assembly strategy consisted of combining short- and long-read sequencing with chromosome conformation capture (Hi-C) technologies. Based on a *k*-mer analysis, the final genome size was estimated to be 2.228 Gb (2.6 Gb with flow cytometry) with a heterozygosity of 5.9%. A total of 71 Gb of SMRT sequences were corrected with small reads and used for the initial contig assembly. After scaffolding and polishing, the total length of the assembly was 1.831 Gb, with a corresponding contig N50 value of 0.382 Mb (Supplementary Table 2). To further improve the assembly, 35.1 Gb of Chicago and 32 Gb of Hi-C library reads were used to anchor, order, and orient the contigs. The final assembly contains 9,019 scaffolds, with a total length of 1.832 Gb and an N50 value of 1.756 Mb (Table 1). About 50% of the total assembled genome was contained in the 262 longest scaffolds. Genome assembly completeness was assessed using the BUSCO with the embryophyta dataset. Of the 1,375 conserved core embryophyta genes used to assess genome completeness, 1,190 (86.6%) of core genes were represented in our genome assembly, compared with other published orchid genomes (Table 1, Supplementary Table 4).

**Table 1.** A comparison between orchid genome assemblies.

	*Phalaenopsis equestris*	*Dendrobium catenatum*	*Phalaenopsis aphrodite*	*Cymbidium sinense^a^*	*Cymbidium goeringii^a^*	*Gongora gibba*
Year	2015	2016	2018	2021	2021	2022
Est. size (Gb)	1.6	1.11	1.2	3.52	4.0	2.23
Assembled size (Gb)	1.09	1.01	1.02	3.45	3.99	1.83
Scaffold N50 (Mb)	0.36	0.39	0.95	NA	NA	1.756
Contig N50 (kb)	20.55	33.09	18.81	1,110	377.6	382.58
Longest scaffold (Mb)	81.76	2.59	10.39	NA	NA	17.67
Repeat content (%)	62	78.1	60.3	77.78	88.87	82.95
BUSCO (%)	91	92.46	95	91	87.8	86.6
No. of genes	29,431	28,910	28,902	29,638	29,556	17,374
No. of scaffolds	236,185	72,901	13,732	20	20	9,019
Reference	Cai *et al.* 2015	Zhang *et al.* 2016	Chao *et al.* 2018	Yang *et al.* 2021	Chung *et al.* 2022	This study

^*a*^Chromosome-level assembly.

The mitochondrial genome was assembled into 13 scaffolds with a total length of 462,164 bp. The size of the plastid genome was 156,794 bp in length including a pair of inverted repeats named IRa and IRb of 26,677 bp that divide the plastid genome into a large single-copy (84,915 bp) and a small single-copy region (18,525 bp). We identified and annotated a total of 114 unique genes, 80 coding sequences, including 21 genes duplicated in the IR region, 30 distinct tRNAs, and 4 distinct rRNA genes.

We confirmed the identity of chemotype A as *G. gibba* by creating a plastid phylogeny with 10 additional *Gongora* samples from Panama. *G. gibba* from Panama and chemotype A from La Gamba, Costa Rica, formed a well-supported clade, and we therefore named chemotype A as *G. gibba* (Supplementary Fig. 2).

### Gene prediction

Using both de novo and library-based repetitive sequence annotation, we annotated 82.95% of the *G.* g*ibba* genome as repeat elements. The repetitive content of *G. gibba* was higher than the most of the other sequenced orchids except for *Cymbidium goeringii* (88.87%; Chung *et al*. 2022). Retrotransposable elements, known to be the dominant form of repeats in angiosperm genomes, constitute a large part of the genome and include the most abundant subtypes, such as LTR/Copia (15.82%), LTR/Ty3 (10.99%; Wei *et al*. 2022), LINE/L1 (0.83%), and LINE/RTE-BovB (1.13%), among others (Supplementary Table 3). Of the repetitive elements, 30.88% could not be classified into any known families, consistent with previous reports from other orchid genomes, suggesting that there may be new repetitive or transposable elements unique to the family Orchidaceae (Zhang *et al*. 2016; Yu *et al*. 2020; Yang *et al*. 2021).

Protein-coding gene models were constructed using a pipeline combining de novo prediction and homology-based prediction methods. In total, 17,374 protein-coding genes were annotated in *G. gibba*. Using the BUSCO to assess the completeness of genic regions using the embryophyta database, we found that 83.6% (1,150/1,375) of orthologous groups were present in annotation (Supplementary Table 4). Furthermore, we assessed annotation quality using the OMArk which assesses not only completeness but also consistency of the annotation compared with the most closely related species available. The OMArk used the Magnoliopsida database to calculate the BUSCO scores, with 88.5% (6,915/7,818) orthologous groups present in the *G. gibba* annotation. Furthermore, 92.0% of the proteome was assessed as having consistent lineage placement, with no contamination (genes whose closest gene families is from another lineage and likely come from contamination), no partial mapping (genes that have <80% of the sequence with shared *k*-mer content from its closest gene family) or fragments (genes with a length less than half the median gene content of its closest gene family) detected. This suggested that both the assembly and annotation quality of the *G. gibba* genome were high.

### Annotation of TPS genes

**Fig. 1.**
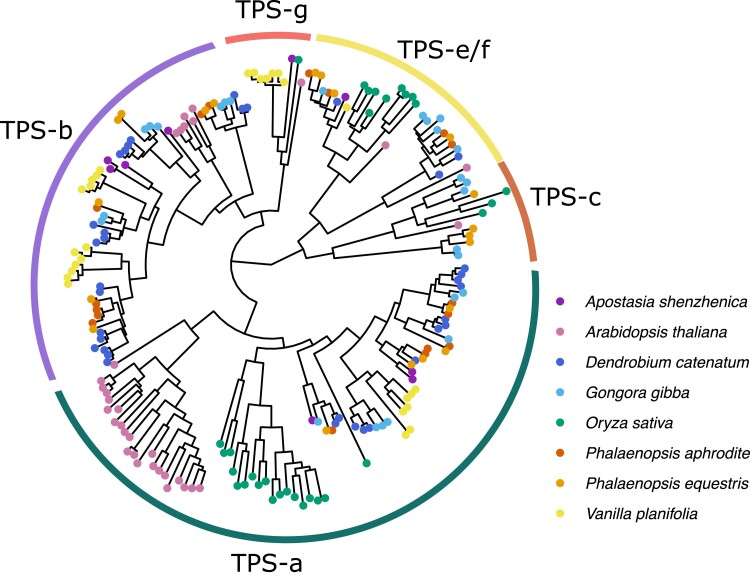
A phylogenetic analysis of flowering plant TPSs. TPS family classifications are illustrated.

We annotated 26 TPS genes in *G. gibba*, similar to the previously described orchid TPS families. To resolve the phylogenetic relationship of the TPS genes and those of other orchids, we constructed a phylogenetic tree based on their amino acid sequences and included TPS gene sequences derived from *A. thaliana* and *O. sativa*, as well as the orchids *Ap. shenzhenica*, *D. catenatum*, *D. officinale*, *P. equestris*, and *V. planifolia.* The 26 putative TPS genes in *G. gibba* were ascribed to the previously recognized TPS subfamilies in angiosperms: TPS-a, TPS-b, TPS-c, and TPS-e/f (Fig. 1). No members of TPS-g were found.

## Discussion

Some orchid species, such as those belonging to the Catasetinae and Stanhopeinae, exhibit highly specialized pollination associations with fragrance-collecting male euglossine bees. In this system, differences in the floral scent profile of closely related lineages can mediate reproductive isolation because floral scent regulates pollinator attraction and specificity (Dressler 1968; Williams and Dodson 1972; Williams and Whitten 1983; Hetherington-Rauth and Ramírez 2016; Guizar Amador 2022). Therefore, the study of speciation in these orchids requires understanding the molecular basis of floral scent emission and the evolutionary forces promoting its differentiation. So far, research in this area has been limited by a lack of genomic resources. In this study, we construct a genome assembly of *G. gibba*, an orchid from the subtribe Stanhopeinae, with an assembled genome size of 1.83 Gb. Alongside the assembly, we present a genome-wide annotation and a high-quality annotation of TPS genes involved in floral scent production. These new resources will facilitate further investigation into the genetic architecture of floral scent and its role in reproductive isolation.

Species identification within the genus *Gongora* poses significant challenges due to little genetic and morphological variation, with cryptic species differentiated by their floral scent (Dressler 1966; Whitten 1985; Jenny 1993; Hetherington-Rauth and Ramírez 2016; Guizar Amador 2022). The genome assembly generated belongs to a group previously treated as chemotype A from La Gamba, Costa Rica (Hetherington-Rauth and Ramírez 2016; Guizar Amador 2022). In this study, we name chemotype A as *G. gibba*. The type specimen of *G. gibba* is from Colon, Panama, a geographically distant location from La Gamba. This species is characterized by specific scent compounds, mainly *trans*-elemicin and *trans*-methyl cinnamate (Whitten 1985). In contrast, chemotype A produces *trans*-methyl-methoxy-cinnamate, estragole, *cis*-methyl-methoxy-cinnamate, and chavicol and attracts different bee pollinators (Hetherington-Rauth and Ramírez 2016). However, plants of *G. gibba* from Panama are morphologically indistinguishable from plants treated as chemotype A from La Gamba, which is confirmed by their close phylogenetic relationship. This chemotype, along with others, is being actively investigated for the presence of cryptic species (Hetherington-Rauth and Ramírez 2016; Guizar Amador 2022). This highlights the need for comprehensive investigations to delineate species boundaries and relationships within *Gongora* chemotypes. However, we emphasize the utility of the genome reported here for bioinformatic studies at the genus level due to the potentially high genetic similarity among *Gongora* species.

A nearly 3,000-fold range of genome sizes has been described in land plants, with larger genomes tending to have higher percentages of transposable elements (Kress *et al*. 2022). Within the flowering plants, Orchidaceae has the most variation in genome size (Leitch *et al*. 2009). The size of the *G. gibba* genome, at 1.83 Gb, is comparable with other sequenced orchids from the subfamily Epidendroideae. The *G. gibba* genome is around half the size of previously sequenced *Cymbidium* genomes and around double that of previously sequenced *Dendrobium* and *Phalaenopsis* genomes. Repeat content is high, 83%, in the *G. gibba* genome, with only *C. goeringii* described as having a higher percentage of repeats at 89%. Given the smaller genome size in *G. gibba* compared with *C. goeringii*, this high percentage of repetitive content is perhaps surprising. Fewer genes were annotated in *G. gibba* compared with other orchids. This could be due to the high ratio of repeats to genome size or due to the stringent criteria used when combining evidence from both RNA-seq and protein homology during annotation.

For decades, biologists have studied plant metabolism, trying to elucidate both the evolutionary function and the underlying genetic basis of plant chemical diversity (Firn and Jones 2003). The plants also exhibit variation in chemical production within species as well as variation between different families or species (van Leur *et al*. 2006; Dussarrat *et al*. 2023). In some species, this variation is so large that chemotypes can be described based on the presence of certain compounds or their ratios. In many cases, the ecological function of these chemotypes and their genetic basis remain unknown. *Gongora* orchids produce a floral scent that attracts their pollinators: male euglossine bees (Allen 1954; Eltz *et al*. 2005). The scent compounds are then collected by the bees, and they use them as a perfume during courtship displays. Different *Gongora* chemotypes produce distinct floral scents, which attract nonoverlapping sets of pollinator species, providing an excellent system to study the evolution of plant chemical diversity (Hetherington-Rauth and Ramírez 2016; Guizar Amador 2022). This study provides the genomic resources needed to make this possible.

Many volatile compounds emitted by *Gongora* flowers are terpenes, a large and structurally diverse class of compounds (Hetherington-Rauth and Ramírez 2016). The diversity of terpenes found in nature is mainly due to the diversification of the TPS gene family that carries out key steps in terpene formation (Tholl 2006). This family of enzymes is present in all land plants and has evolved rapidly through gene duplication and sequence divergence, resulting in an astounding diversity of often lineage-specific terpene compounds (Chen *et al*. 2011). The total number of TPS genes in a genome differs between species, and in orchids, they have been found to range from 14 in *Ap. shenzhenica* (Yu *et al*. 2020) to 48 in *Dendrobium chrysotoxum* (Zhang *et al*. 2021). TPS genes are classified into 7 subfamilies: TPS-a, TPS-b, TPS-c, TPS-d, TPS-e/f, TPS-g, and TPS-h (Chen *et al*. 2011). In the *G. gibba* genome, we identified and annotated 26 different TPS genes belonging to multiple subfamilies. Based on the previously identified chemotypes (Hetherington-Rauth and Ramírez 2016), we expect *G. gibba* (chemotype A) to be able to produce both monoterpene and sesquiterpenes (terpenes of different sizes). We find that *G. gibba* has 6 TPSs from the TPS-e/f subfamily and 8 from the TPS-b subfamily, both shown to be involved in monoterpene biosynthesis in the floral tissue of *Phalaenopsis bellina* (Huang, Kuo, *et al*. 2021). Furthermore, we identified 7 TPSs belonging to the TPS-a family, most of which have been described as sesquiterpene synthases (Chen *et al*. 2011). Lineage-specific expansions are present in all 3 subfamilies (TPS-a, TPS-b, and TPS-e/f), highlighting the dynamic nature of the evolution of this gene family.

Changes in the number of TPS genes, or in their coding sequences, are not the only mechanisms for scent differentiation. In fact, especially among more closely related lineages, we expect differences in floral scent to be driven by differential expression and regulatory mechanisms upstream of biosynthetic pathways underlying the production of volatile organic compounds. We propose that *Gongora* is an excellent system for studying the rapid evolution of floral scent, particularly those changes involving different biosynthetic pathways. For example, *G. gibba* (chemotype A) and chemotype M from La Gamba are closely related to each other, occur sympatrically and have overlapping flowering phenologies. Through pollinator network reconstruction, we have previously shown that each chemotype attracts a unique set of pollinator species, but reproductive isolation is not complete (Guizar Amador 2022). Despite the occurrence of gene flow, no plants with intermediate floral phenotypes have been observed so far, suggesting that hybridization is rare or that selection against intermediate phenotypes is strong. Not only are the floral scents different between the 2 chemotypes, but the volatile compounds emitted by these 2 lineages are the products of 2 unrelated biosynthetic pathways: *G. gibba* (chemotype A) produces mainly aromatic compounds and chemotype M emits monoterpenoids (Hetherington-Rauth and Ramírez 2016; Guizar Amador 2022). Due to the close evolutionary relationship between the 2 chemotypes, we expect that identifying differentially expressed genes in the labellum of these orchids will shed light on the regulatory networks involved in differential floral scent biosynthesis.

### Conclusion

We generated a high-quality reference genome for *G. gibba* which will serve as a crucial resource for understanding the evolution and maintenance of reproductive barriers in euglossine bee–pollinated orchids. Future studies may focus on elucidating the molecular mechanisms that control pollinator specialization in this group by investigating the expression and regulation of biosynthetic pathways involved in floral scent production.

## Supplementary Material

jkae211_Supplementary_Data

## Data Availability

All raw sequence data and the genome assembly were deposited to the NCBI under the BioProject accession no. PRJNA1014482. The raw RNA-seq data used for genome annotation were deposited in the SRA under the BioProject accession no. PRJNA1027883 with accession nos. SAMN37807278–SAMN37807288. The plastid sequences were deposited under the BioProject no. PRJNA1146482. Notebooks with parameter details, scripts for phylogenetic analyses, and the genome annotation are available at the OSF (DOI 10.17605/OSF.IO/HQAV3). Supplemental material available at G3 online.
