# Supplementary material for: The Gongora gibba genome assembly provides new insights into the evolution of floral scent in male euglossine bee–pollinated orchids
Source: G3 (Bethesda). 2024 Sep 4;14(11):jkae211. doi: 10.1093/g3journal/jkae211 (PMC11540329; doi:10.1093/g3journal/jkae211)
Supplement: jkae211_Supplementary_Data [file jkae211_supplementary_data.zip › Supplemental_Material_G3-2024-405171.docx]

A


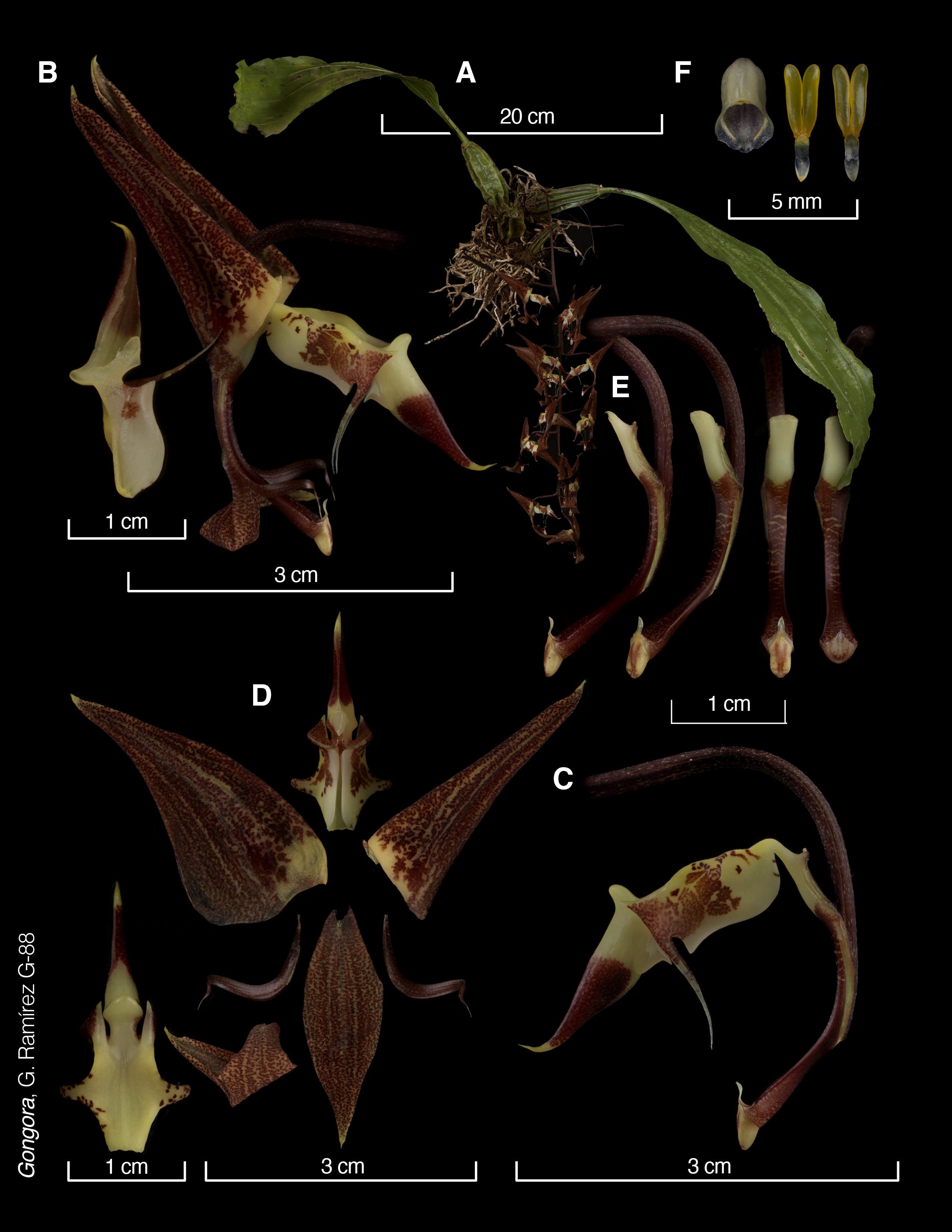

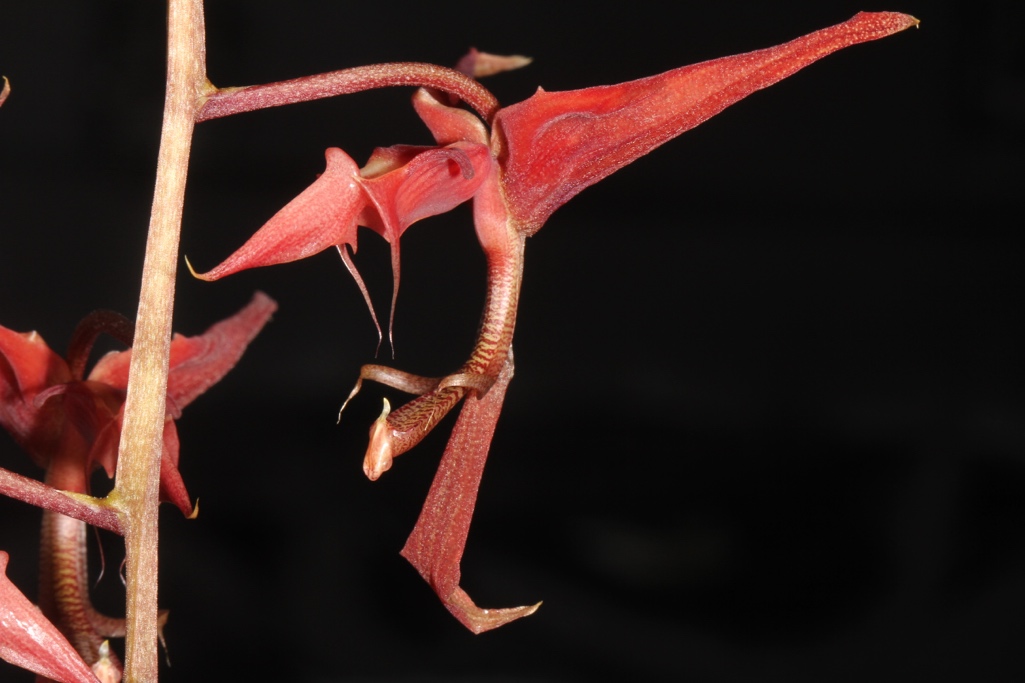


B

Figure S1. A) Composite plate of *Gongora gibba* (chemotype A). A. Plant. B. Flower. C. Ovary, column and lip, lateral view. E.  Dissected perianth. E. Column, lateral, and front views. F. Pollinarium and anther cap. Based on SRG-88 (JBL). Photographs and plate by Franco Pupulin. B) *Gongora gibba* individual G-10 used for genome sequencing. Photograph by Santiago Ramírez.


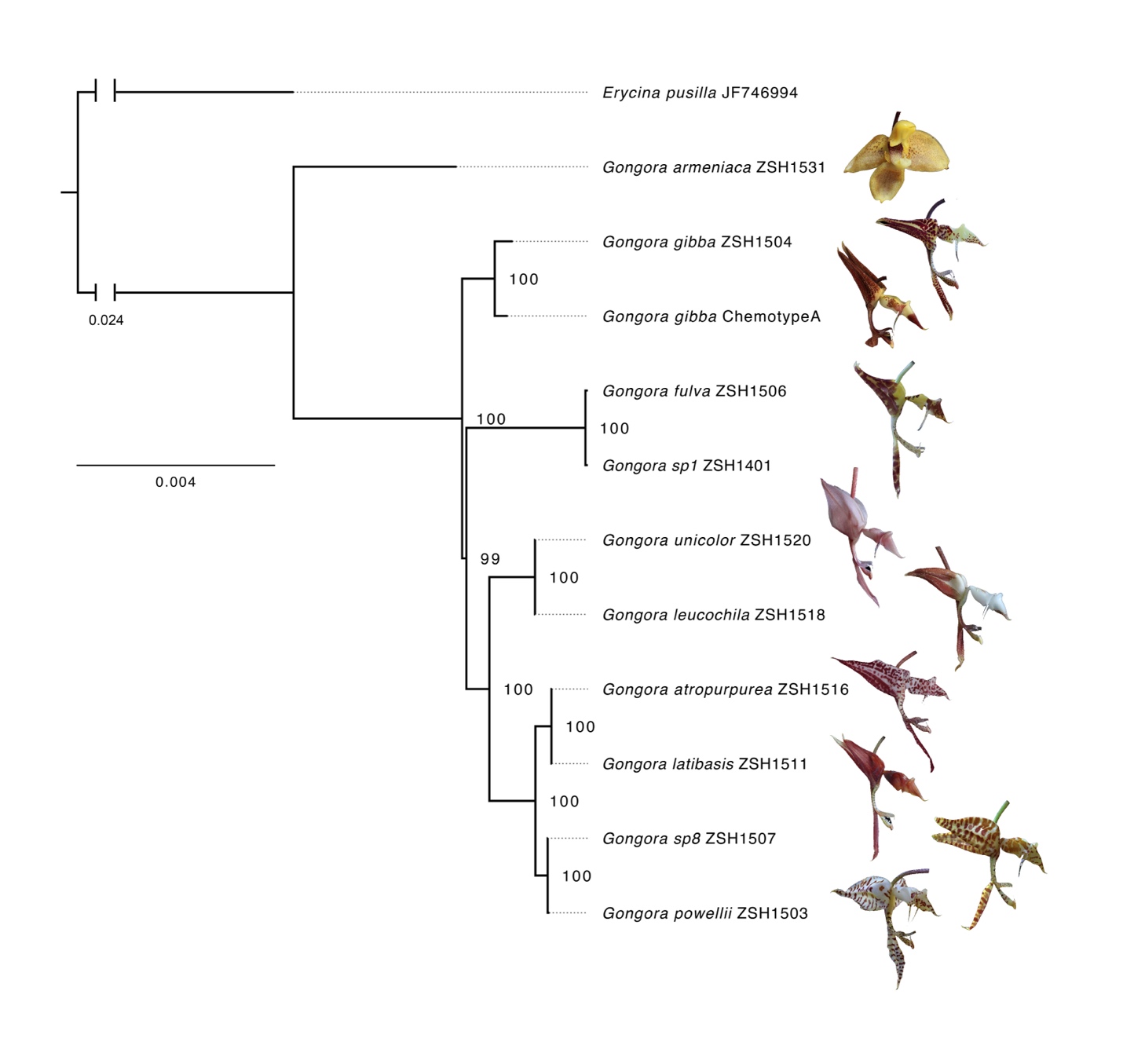


Figure S2. Plastid phylogeny illustrating the placement of *Gongora gibba* ChemotypeA (genome sequenced in this study) in the same clade as previously identified *Gongora gibba* from Panama.

Table S1 Coverage statistics for Gongorav1.sp

|  | Gongorav1.srp Coverage |
| --- | --- |
| Mean coverage | 94.57 |
| Standard deviation | 489.84 |
| Minimum | 0 |
| 25% | 40 |
| 50% | 50 |
| 75% | 62 |
| Maximum | 417435 |

Note. Summary statistics for the genomic coverage of Gongorav1.srp using the SRCD2 S1 L001 library.

Table S2 Genome assembly statistics

|  | Gongorav1.pil.fa | Gongorav1.1 |
| --- | --- | --- |
| N50 | 382,585 | 1,756,489 |
| L50 | 1,308 | 262 |
| Total length (Gbp) | 1.831 | 1.833 |
| GC% | 32.6 | 32.6 |
| Number of Ns per 100 kbp | 0.09 | 73.04 |
| Longest contig/scaffold (Mbp) | 3.02 | 17.68 |
| Number of contigs/scaffolds | 17,920 | 9,019 |

Table S3 Repetitive sequences identified in the *Gongora* genome

|  | Number of elements* | Length occupied (bp) | % of sequence |
| --- | --- | --- | --- |
| **Retroelements (Total)** | 468828 | 580225206 | **31.66** |
| Total LINEs | 47017 | 35868914 | 1.96 |
| LINEs (RTE/Bov-B) | 36281 | 20718697 | 1.13 |
| LINEs (L1/CIN4) | 10642 | 15145735 | 0.83 |
| Total LTRs | 421811 | 544356292 | 29.7 |
| LTR (Ty1/Copia) | 200937 | 289956828 | 15.82 |
| LTR (Ty3/DIRS1) | 138322 | 201491459 | 10.99 |
| **DNA transposons (Total)** | 1095483 | 344136276 | **18.78** |
| hobo-Activator | 9377 | 4291872 | 0.23 |
| MULE-MuDR | 3209 | 2561920 | 0.14 |
| Tourist-Harbinger | 6590 | 4387442 | 0.24 |
| **Rolling-circles** | 1916 | 1468403 | **0.08** |
| **Unclassified** | 916776 | 565956361 | **30.88** |
| **Total interspersed repeats** |  | 1490317843 | **=81.31** |
| **Simple repeats** | 351159 | 26142710 | 1.43 |
| **Low complexity** | 44808 | 2482357 | 0.14 |
| **Total repeats masked** |  |  | **82.95** |

* Most repeats fragmented by insertions or deletions have been counted as one element

Table S4 BUSCO results for both genome and annotation searched against the Embryophyta database

|  | Genome | Annotation |
| --- | --- | --- |
| Complete | 1190 (86.6%) | 1150 (83.6%) |
| Complete and single copy | 1170 (85.1%) | 960 (69.8%) |
| Complete and duplicated | 20 (1.5%) | 190 (13.8%) |
| Fragmented | 76 (5.5%) | 52 (3.8%) |
| Missing | 109 (7.9%) | 173 (12.6%) |
| Total searched | 1375 | 1375 |
